# Use of Mobile Apps for Visual Acuity Assessment: Systematic Review and Meta-analysis

**DOI:** 10.2196/26275

**Published:** 2022-02-14

**Authors:** Lingge Suo, Xianghan Ke, Di Zhang, Xuejiao Qin, Xuhao Chen, Ying Hong, Wanwei Dai, Defu Wu, Chun Zhang, Dongsong Zhang

**Affiliations:** 1 Department of Ophthalmology Peking University Third Hospital Beijing China; 2 Department of Ophthalmology Qilu Hospital of Shandong University Jinan China; 3 Department of Business Information Systems and Operations Management University of North Carolina at Charlotte Charlotte, NC United States

**Keywords:** smartphone, iPad, eye screening, visual acuity, app, meta-analysis

## Abstract

**Background:**

Vision impairments (VIs) and blindness are major global public health issues. A visual acuity (VA) test is one of the most crucial standard psychophysical tests of visual function and has been widely used in a broad range of health care domains, especially in many clinical settings. In recent years, there has been increasing research on mobile app–based VA assessment designed to allow people to test their VA at any time and any location.

**Objective:**

The goal of the review was to assess the accuracy and reliability of using mobile VA measurement apps.

**Methods:**

We searched PubMed, Embase, Cochrane Library, and Google Scholar for relevant articles on mobile apps for VA assessment published between January 1, 2008, and July 1, 2020. Two researchers independently inspected and selected relevant studies. Eventually, we included 22 studies that assessed tablet or smartphone apps for VA measurement. We then analyzed sensitivity, specificity, and accuracy in the 6 papers we found through a meta-analysis.

**Results:**

Most of the 22 selected studies can be considered of high quality based on the Quality Assessment of Diagnostic Accuracy Studies–2. In a meta-analysis of 6 studies involving 24,284 participants, we categorized the studies based on the age groups of the study participants (ie, aged 3-5 years, aged 6-22 years, and aged 55 years and older), examiner (ie, professional and nonprofessional examiners), and the type of mobile devices (ie, smartphone, iPad). In the group aged 3 to 5 years, the pooled sensitivity for VA app tests versus clinical VA tests was 0.87 (95% CI 0.79-0.93; *P*=.39), and the pooled specificity was 0.78 (95% CI 0.70-0.85; *P*=.37). In the group aged 6 to 22 years, the pooled sensitivity for VA app tests versus clinical VA tests was 0.86 (95% CI 0.84-0.87; *P*<.001), and the pooled specificity for VA app tests versus clinical VA tests was 0.91 (95% CI 0.90-0.91; *P*=.27). In the group aged 55 years and older, the pooled sensitivity for VA app tests versus clinical VA tests was 0.85 (95% CI 0.55-0.98), and the pooled specificity for VA app tests versus clinical VA tests was 0.98 (95% CI 0.95-0.99). We found that the nonprofessional examiner group (AUC 0.93) had higher accuracy than the professional examiner group (AUC 0.87). In the iPad-based group, the pooled sensitivity for VA app tests versus clinical VA tests was 0.86, and the pooled specificity was 0.79. In the smartphone-based group, the pooled sensitivity for VA app tests versus clinical VA tests was 0.86 (*P*<.001), and the pooled specificity for VA app tests versus clinical VA tests was 0.91 (*P*<.001).

**Conclusions:**

In this study, we conducted a comprehensive review of the research on existing mobile apps for VA tests to investigate their diagnostic value and limitations. Evidence gained from this study suggests that mobile app–based VA tests can be useful for on-demand VI detection.

## Introduction

Vision impairments (VIs) and blindness are a major global public health issue [1]. In 2020, the estimated number of people with distance VI in the world was 596 million, including 43 million with blindness [2]. A large proportion of those affected (90%) live in low- and middle-income countries. VI can be preventable or treatable for approximately 90% of people with VI by using highly cost-effective interventions. In low-income countries, diagnosis, monitoring, and treatment of vision problems are challenging, largely attributable to insufficient eye care professionals [3]. In high-income countries, there are also barriers to eye screening and patient compliance. In particular, there is often time pressure in primary consultations for diagnosing ophthalmic problems [4]. Thus, there is a need for ubiquitous, self-manageable, and automated tools for visual acuity (VA) tests to increase early detection and timely assistance for people with VIs [5].

To address the lack of eye care professionals and reduce the cost for eye screening, an increasing amount of research effort has been dedicated for building efficient eye screening tools and methods by leveraging mobile devices (eg, smartphones) and technologies [6]. In both high- and low-income countries, the continuous growth of mobile device ownership has propelled mobile health (mHealth) interventions [7]. Mobile technologies provide point-of-care tools for real-time patient monitoring, patient data collection, health information delivery, and telemedicine throughout the world [8]. Free and paid mHealth apps have demonstrated notable success in detecting ophthalmic diseases [9], and the number of mobile apps intended to address eye care issues has been increasing. The VA test, a vision test often performed by an optometrist or ophthalmologist to measure a person’s ability to see an object from 20 feet away, is one of the most crucial standard psychophysical tests for assessing visual function [10]. It is also a measurement of eye treatment effectiveness and changes in central vision over time in clinical settings [11].

Traditional clinical ophthalmic equipment is cumbersome and difficult to transport, lacks mobility, and requires trained ophthalmic professionals. Therefore, an automated, accurate, and user-friendly approach is needed for vision screening or self-monitoring. Some studies have proposed novel mobile device–based techniques for a VA test [12]. Bastawrous et al [13] conducted the Early Treatment Diabetic Retinopathy Study (ETDRS) that proposed the Peek Acuity mobile app, which was validated against Snellen charts. ETDRS charts were used as part of a survey about epidemiologic eyes among adults in central Kenya. Peek Acuity is a Logarithm of the Minimum Angle of Resolution (logMAR)-style smartphone-based vision test. Using a fast-testing algorithm, it is capable of measuring VA at a clinically acceptable time, with greater reliability and precision than those using Snellen charts [14]. It also allows individuals to choose from multiple types of visual charts for VA assessment, such as the Snellen [15], ETDRS [16], and Tumbling E [17] charts. Thus, the Peek Acuity app provides an advantage over traditional logMAR acuity measurement.

Although current modern mobile devices with high-resolution screens offer novel, ubiquitous, and portable vehicles for VA tests, it remains unclear whether existing VA test apps are effective for ophthalmic disease diagnosis and management. In this study, we conducted a comprehensive review of the research to investigate the diagnostic accuracy and limitations of existing mobile VA assessment apps for detecting VI. Based on age, test examiner, and type of mobile devices, we categorized existing studies into different categories and performed subgroup analysis. Furthermore, 4 variables (ie, publication year, sample size, mobile device, and examiner) were selected in the multivariate meta-regression.

## Methods

### Data Sources and Search Strategy

We searched PubMed, Embase, Cochrane Library, and Google Scholar for relevant articles on mobile apps for VA testing published between January 1, 2008, and July 1, 2020. The literature search used the following terms, as well as their different combinations, as the search keywords: smartphone, iPhone, iPad, phone, tablet, mobile devices, visual acuity, VA, eye screening, app, application, Snellen chart, Tumbling E chart, Early Treatment Diabetic Retinopathy Study chart, and ETDRS chart (Multimedia Appendix 1).

### Inclusion and Exclusion Criteria

We applied several inclusion criteria when identifying relevant studies for this research. A study would be considered relevant if it (1) was to evaluate VA via a smartphone or tablet app, (2) used an acceptable VA reference standard, (3) was written in English, and (4) was published after 2008. Exclusion criteria included (1) studies where the number of participants with VI was fewer than 10 and (2) literature review or commentary articles, short communications, or case reports.

### Data Collection

Two authors and a research assistant extracted information from the identified studies, including the study design, sample size, participant characteristics, nature of eye screening, mobile techniques (eg, functions and features of smartphones and tablet app), and main research results (eg, true positives, false positives, true negatives, and false negatives). The extracted information was reviewed and verified by other coauthors.

### Risk of Bias and Quality Assessment

Two researchers specializing in eye care independently reviewed each selected article and assessed its quality by using the Quality Assessment of Diagnostic Accuracy Studies–2 scores (QUADAS-2) tool. They discussed and resolved disagreements in their scores with other coauthors through a face-to-face meeting. Among included studies, the risk of bias was evaluated in 4 aspects by the QUADAS-2 tool: patient selection, index test, reference standard, and flow and timing. For applicability concerns, we assessed patient selection, index test, and reference standard as low, high, or unclear.

### Statistical Analysis

We used chi-square and *I*^2^ values of sensitivity, specificity, likelihood ratio tests, and diagnostic odds ratio (DOR) to evaluate heterogeneity. Heterogeneity was evaluated by Cochrane Q-test (*I*^2^ value); heterogeneity was considered to exist when *P*>.10. When *I*^2^ results were ≤50%, a fixed effects model was used; otherwise, a random effects model was used. The values of DOR ranges from 0 to infinity, with 0 indicating no test discrimination. Higher scores indicate better discrimination. The sensitivity, specificity, likelihood ratio positive (LR+), likelihood ratio negative (LR–), and DOR of each age, examiner, and mobile device type subgroup were calculated using a random effects model given the high expected heterogeneity. We performed meta-regression to explore whether the sources of heterogeneity could be explained by some methodological factors (eg, year of publication, sample size, mobile device, examiner) and characteristics of study samples. We also constructed a summary receiver operating characteristic (SROC) curve using the Moses constant of linear mode model. We used Meta-DiSc software (version 1.4, Ramón y Cajal Hospital, Madrid, Spain) for meta-analysis and Review Manager (version 5.3, Cochrane Collaboration) for paper quality assessment. Extracted data were synthesized by creating forest plots of sensitivity and specificity.

## Results

### Search Results

**Figure 1:**
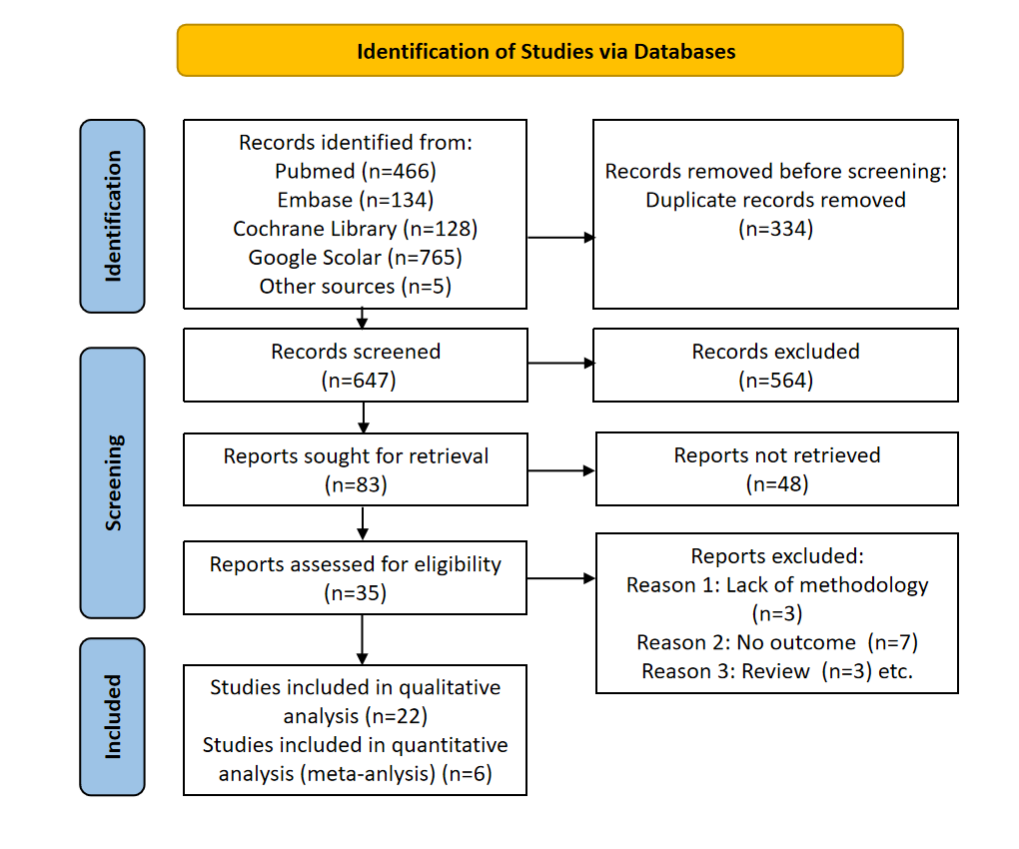
Flowchart of systematic literature search.

Our literature search yielded a total of 981 papers. After our review of the titles and abstracts, 959 studies were excluded either because of duplication or lack of adherence to our topic, resulting in 22 full-text articles for quality assessment (Figure 1). We also checked their reference lists for further relevant studies and retrieved additional studies. Finally, 6 full-text studies met inclusion criteria for quantitative analysis (meta-analysis). Figure 1 presents the flowchart of our systematic literature search. The selected studies were conducted in more than 11 countries.

### Characteristics of the Studies

Among the 22 identified studies, 4 studies were population-based, 10 were observational, 3 were cross-sectional, 4 were prospective, and 1 was a validation study (Table S1 in Multimedia Appendix 2 and Multimedia Appendix 3). Nine studies assessed the performance of tablet-based (ie, iPad) VA measurement apps, and 13 studies assessed the performance of smartphone-based apps. A total of 25 mobile apps were evaluated in those 22 enrolled studies.

### Characteristics of Study Samples

Among the 22 identified studies, the average number of participants per study was 1648, ranging from 43 [18] to 10,579 participants [17]. The age of the participants in those studies varied significantly from 3 to 89 years. Five studies included children aged younger than 18 years [17,19-22], 10 studies included middle-aged (19-55 years) adults [5,9,12,18,23-28], and the remaining 7 studies included older adults (aged 55 years and older) [7,13,14,29-31]. The main demographic characteristics of the participants in each study are summarized in Table S1 in Multimedia Appendix 2 and Multimedia Appendix 3.

### Eye Screening with VA apps

Among the 22 selected papers, 9 studies used iPads for VA tests [5,18,21,23,28-30,32,33], and 13 used smartphones [7,9,12-14,17,19,20,22,24,25,27,31]. Six studies used the Peek Acuity app (Tumbling E chart) [7,13,17,19,20,22], 2 used the Sightbook app (Snellen chart) [31,32], 2 used the Eye Chart Pro app (Snellen chart) [5,28], and 1 [12] evaluated 11 different VA apps (eg, Eye Test app, OptOK app). The rest of the studies investigated other VA apps.

A total of 10 studies used the Tumbling E chart in mobile apps. A Tumbling E chart, also known as an E chart, is useful for patients who are unable to read the Latin alphabet (eg, very young children). The chart contains multiple rows of the letter E in various rotations and with decreasing sizes. Patients were asked to state where the limbs of the E were pointing (up, down, left, or right). Depending on how far a patient can see, his or her VA can be quantified. The Tumbling E chart shares the same principle as the Snellen distant vision chart [7,9,13,17-20,22,28,30].

Three studies used the ETDRS chart for VA measurement in mobile apps. A logMAR chart, also called a Bailey-Lovie chart or an ETDRS chart, is a chart consisting of rows of letters used by ophthalmologists, orthoptists, optometrists, and vision scientists to estimate VA. The chart was developed by the National Vision Research Institute of Australia in 1976 to enable a more accurate estimate of VA than other charts (eg, the Snellen chart). For this reason, the logMAR chart has been recommended, particularly in a research setting [14,23,29].

Five studies used the Snellen chart in VA measurement apps. A Snellen chart is another eye chart used to measure VA. Snellen charts are named after the Dutch ophthalmologist Herman Snellen, who developed the chart in 1862. The normal Snellen chart is printed with eleven lines of block letters, also known as optotypes. The first line consists of a single very large letter. Subsequent rows have increasing numbers of letters that decrease in size. A person taking the test covers one eye from 6 meters or 20 feet away and reads the letters of each row from the top to the bottom. The rest of the studies used other types of charts for VA measurement, such as Landolt C or Numbers [5,12,18,27,32].

The 22 selected papers measured VA at a range from 36 centimeters to 6 meters: 8 studies measured VA at a 2-meter testing distance [13,14,19,20,29]; 3 studies measured VA at a 3-meter testing distance [9,21,26]; and the remaining 11 studies measured VA at 36 cm, 40 cm, 1 m, 1.2 m, 2 m, 14 inches, 20 feet, 4 m, and 6 m.

### Meta-analysis

Table 1 shows the characteristics and findings of 6 meta-analyses on mobile apps for VA testing. Table 2 shows the summary of 6 meta-analyses that examined mobile apps for VA testing. We categorized studies based on the age of their participants, examiner, and the type of mobile devices. As summarized in Multimedia Appendix 4, 4 variables (ie, publication year, sample size, mobile device, examiner) were selected in the multivariate meta-regression (sensitivity); however, none of those variables was significantly associated with the detected heterogeneity, as shown in Multimedia Appendix 5.

**Table 1:** Main characteristics and findings of 6 meta-analyses.

Source	Study design	Age, year	Sample size (P/E^a^)	Mobile device type	App name	App description	TD^b^	Main results
Nik Azis et al [21], Malaysia	Cross-sectional study	5-6	195/290	iPad mini	AAPOS^c^ Vision Screening	Lea symbols chart	3 m	Sensitivity: 82.1% (right vision), 82.1% (left vision); Specificity: 81.3% (right vision), 76.9% (left vision)
Rono et al [17], Kenyan	Population-based study	11.5, 11.7	S^d^:10,284/S:20,568, P^e^:10,579/P:21,158	Samsung Galaxy S3	Peek Acuity	Tumbling E chart	2 m	Sensitivity: 76.9% (64.8%-86.5%), Specificity: 90.8% (89.3%-92.1%)
Zhao et al [22], US	Prospective study	3-17	106/212	Samsung Galaxy S3 SGH-i747	Peek Acuity	Tumbling E chart	2 m	Sensitivity: 83%-86% for decreased vision, Sensitivity: 69%-83% for referable ocular disease
de Venecia et al [20], US	Observational study	6-17	393/190	Samsung Galaxy A3	Peek Acuity	Tumbling E chart	2 m	Sensitivity: 48%, Specificity: 83%
Bastawrous et al [13], UK	Population-based study	55-97	233/466	Galaxy S3 GT-I9300	Peek Acuity	Tumbling E chart	2 m	Sensitivity: 84.6% (95% CI 54.5%-97.6%); Specificity: 97.7% (95% CI 94.8%-99.3%)
Andersen et al [19], Botswana	Population-based study	6-22	12,877/–	Android phones	Peek Vision	Tumbling E chart	2 m	Sensitivity: 91.6%; Specificity: 90.7%

^a^P/E: participant/eye.

^b^TD: test distance.

^c^AAPOS: American Association for Pediatric Ophthalmology and Strabismus.

^d^S: standard group.

^e^P: peek group.

**Table 2:** A summary of mobile apps for evaluating visual acuity.

Types			Sensitivity		Specificity		Positive LR^a^		Negative LR		Diagnostic OR^b^		AUC^c^
**Examiners**													
	Professional	0.72 (0.66-0.79)		0.80 (0.71-0.85)		3.81 (2.87-5.06)		0.30 (0.10-0.90)		12.25 (4.33-34.71)		0.87 (0.83-0.91)	
	Nonprofessional	0.87 (0.85-0.89)		0.91 (0.90-0.91)		8.66 (8.62-10.98)		0.17 (0.08-0.34)		54.60 (21.98-135.59)		0.93 (0.86-1.00)	
**Patient age (years)**													
	3-5	0.87 (0.79-0.93)		0.78 (0.70-0.85)		3.93 (2.82-5.46)		0.17 (0.10-0.28)		24.01 (11.95-48.22)		—^d^	
	6-22	0.86 (0.84-0.87)		0.91 (0.90-0.91)		8.04 (6.49-9.98)		0.25 (0.10-0.66)		25.47 (9.02-71.94)		0.96 (0.92-0.99)	
	≥55	0.85 (0.55-0.98)		0.98 (0.95-0.99)		37.23 (15.18-91.30)		0.16 (0.04-0.56)		236.50 (41.17-1358.49)		—	
**Mobile devices**													
	iPads	0.86 (0.76-0.92)		0.79 (0.71-0.86		4.14 (2.85-6.01)		0.18 (0.11-0.31)		22.96 (10.69-49.30)		—	
	Smartphones	0.86 (0.84-0.87)		0.91 (0.90-0.91)		8.01 (6.21-10.32)		0.23 (0.09-0.54)		33.86 (13.02-88.06)		0.92 (0.81-1.00)	

^a^LR: likelihood ratio.

^b^OR: odds ratio.

^c^AUC: area under the curve.

^d^Not applicable.

### Meta-analysis of Mobile Apps for VA Testing With Different Age Groups

**Figure 2:**
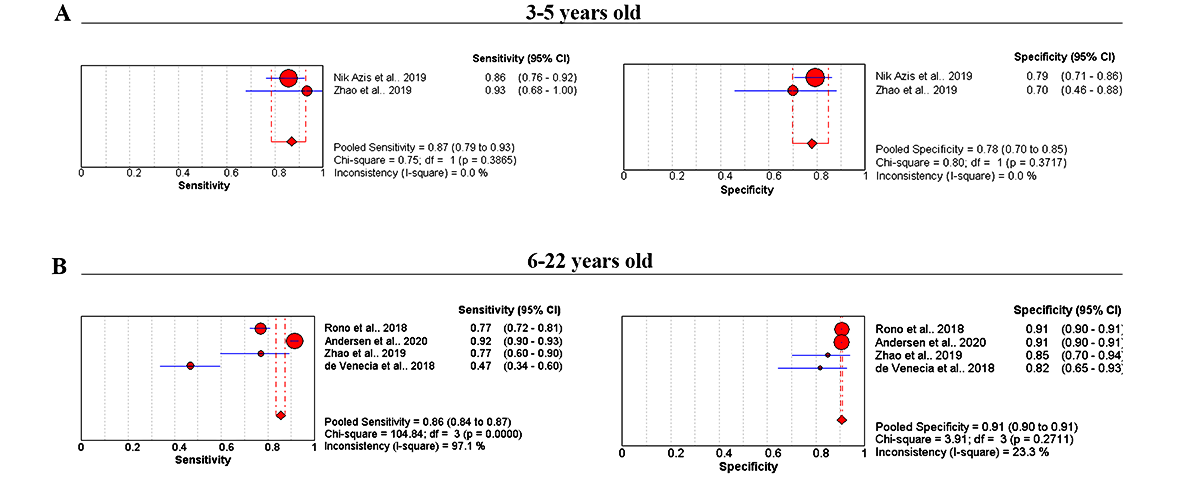
Summary of sensitivity and specificity of meta-analysis studies with different age groups: (A) age 3-5 years (230 participants) and (B) age 6-22 years (23,626 participants).

The 6 meta-analyses involved 24,089 participants. For the group aged 3 to 5 years (230 participants), we used a fixed effects model. The pooled sensitivity was 0.87 (95% CI 0.79-0.93; *P*=.39), and the pooled specificity was 0.78 (95% CI 0.70-0.85; *P*=.37; Figure 2A). LR+ was 3.93 (95% CI 2.82-5.46), LR– was 0.17 (95% CI 0.10-0.28), and DOR was 24.01 (95% CI 11.95-48.22).

**Figure 3:**
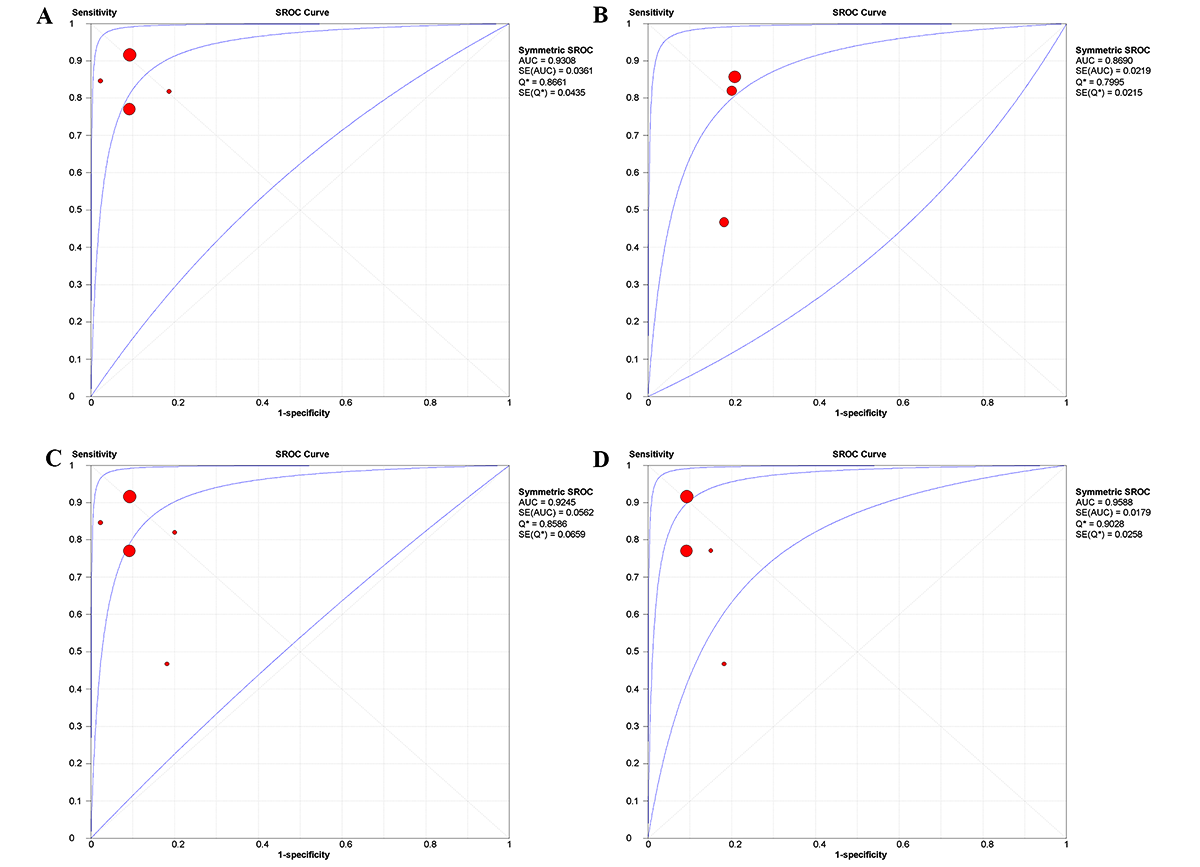
Summary receiver operating characteristic curves for study groups included in the meta-analysis: (A) nonprofessional examiners, (B) professional examiners, (C) smartphone-based, and (D) age 6-22 years.

For the group aged 6 to 22 years (23,626 participants), a random effects model was chosen within these studies because of the significant heterogeneity (*P*<.10, *I*^2^>50%). The pooled sensitivity was 0.86 (95% CI 0.84-0.87; *P*<.001), and the pooled specificity was 0.91 (95% CI 0.90-0.91; *P*=.27; Figure 2B). LR+ was 8.04 (95% CI 6.49-9.98), LR– was 0.25 (95% CI 0.10-0.66), DOR was 25.47 (95% CI 9.02-71.94), and AUC (area under the curve) was 0.96 (95% CI 0.92-0.99; Figure 3D).

In the group aged 55 years and older (233 participants), we used a random effects model. The pooled sensitivity was 0.85 (95% CI 0.55-0.98), and the pooled specificity was 0.98 (95% CI 0.95-0.99). LR+ was 37.23 (95% CI 15.18-91.30), LR– was 0.16 (95% CI 0.04-0.56), and DOR was 236.50 (95% CI 41.17-1358.49).

### Meta-analysis of Mobile Apps for VA Testing With Different Examiners

**Figure 4:**
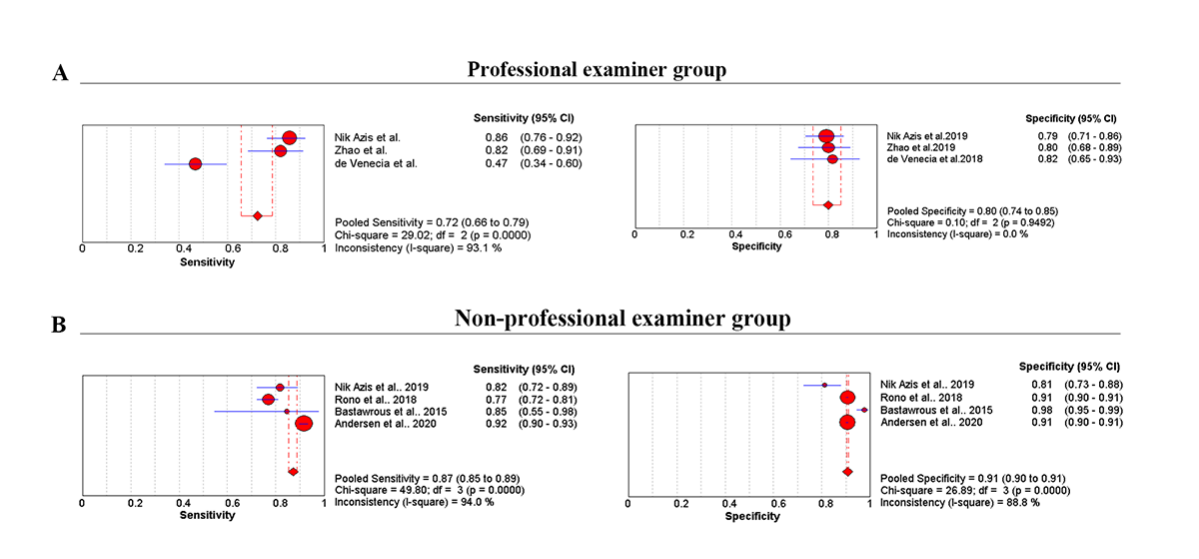
Summary of sensitivity and specificity of the meta-analysis studies with different examiner groups: (A) professional examiners and (B) nonprofessional examiners.

Data from 24,284 participants were analyzed in the 6 meta-analyses. For the professional examiner group (400 participants), we deployed a fixed effects model. The pooled sensitivity was 0.72 (95% CI 0.66-0.79; *P*<.001), and the pooled specificity was 0.80 (95% CI 0.71-0.85; *P*=.95; Figure 4A). LR+ was 3.81 (95% CI 2.87-5.06), LR– was 0.30 (95% CI 0.10-0.90), DOR was 12.25 (95% CI 4.33-34.71), and AUC was 0.87 (95% CI 0.83-0.91; Figure 3B).

In the nonprofessional examiner group (23,884 participants), we deployed a random effects model. The pooled sensitivity was 0.87 (95% CI 0.85-0.89; *P*<.001), and the pooled specificity was 0.91 (95% CI 0.90-0.91; *P*<.001; Figure 4B). LR+ was 8.66 (95% CI 8.62-10.98), LR– was 0.17 (95% CI 0.08-0.34), DOR was 54.60 (95% CI 21.98-135.59), and AUC was 0.93 (95% CI 0.86-1.00; Figure 3A).

### Meta-analysis of Mobile Apps for VA Testing With Different Mobile Devices

Data from 24,284 participants were analyzed in the 6 studies, which used mobile apps either on iPads or smartphones. We used a random effects model for both iPad and smartphone groups. In the iPad-based group (195 participants), the pooled sensitivity of eyes was 0.86 (95% CI 0.76-0.92), and the pooled specificity was 0.79 (95% CI 0.71-0.86). LR+ was 4.14 (95% CI 2.85-6.01), LR– was 0.18 (95% CI 0.11-0.31), and DOR was 22.96 (95% CI 10.69-49.30).

**Figure 5:**
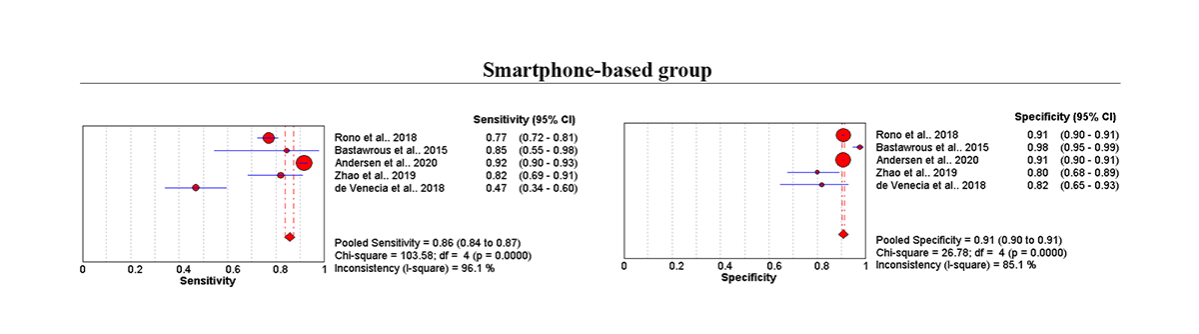
Summary of sensitivity and specificity of the smartphone-based group included in the meta-analysis.

In the smartphone-based group (24,089 participants), the pooled sensitivity was 0.86 (95% CI 0.84-0.87; *P*<.001), and the pooled specificity was 0.91 (95% CI 0.90-0.91; *P*<.001; Figure 5). LR+ was 8.01 (95% CI 6.21-10.32), LR– was 0.23 (95% CI 0.09-0.54), DOR was 33.86 (95% CI 13.02-88.06), and AUC was 0.92 (95% CI 0.81-1.00; Figure 3C).

### Study Quality Assessment

**Figure 6:**
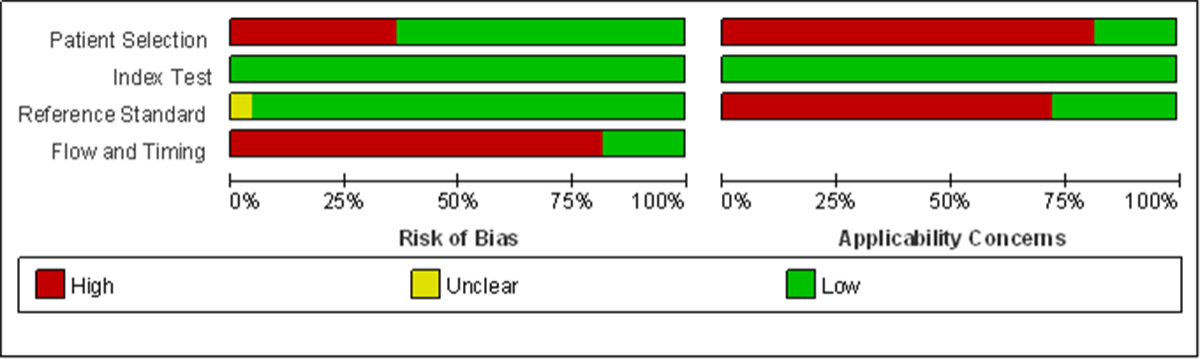
Risk of bias and applicability concerns of studies included in the literature review.

**Figure 7:**
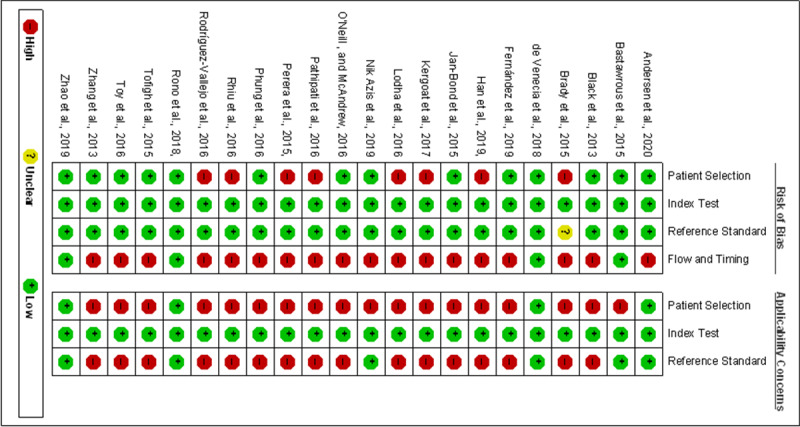
Quality of the included studies assessed via the Quality Assessment of Diagnostic Accuracy Studies–2 tool.

We assessed the quality of the 22 included studies by using the QUADAS-2 tool (Figures 6 and 7). Most studies were of high quality with low risk of bias and applicability concerns. Because of the nature of mobile apps, participant blinding was not always feasible in trials. We considered studies at high risk of bias when they involved participants without clear diagnosis of VI or when there was no strict standardization in the VA examination process and examination conditions.

## Discussion

### Principal Findings

With increasing smartphone and tablet penetration, mobile VA apps provide a good quality, repeatable, objective, and cost-effective approach to vision test for eye screening. For low-income countries, with most of the world’s blind people, the effective tools and techniques to improve early detection and appropriate referral are critical to reducing VI [19]. A VA test is fundamental to evaluating visual function. Accurate assessment of VA depends heavily on factors such as viewing distance, chart illumination, type of eye chart used, and scoring technique used [23].

Mobile devices, which are portable and equipped with a high-resolution screen, provide a novel platform for VA testing. An increasing number of mobile apps for VA testing have been developed that can be downloaded to different mobile platforms. Most of them, however, have not been evaluated for accuracy and reliability for following a reference standard. In our study, we aimed to systematically review and evaluate the accuracy of mobile apps for VA testing. In general, our analysis reveals that those apps investigated in the selected studies performed well in VA testing. They had different levels of accuracy for different participant age groups, between professional and nonprofessional examiners, and between apps for iPad and apps for smartphones. We observed increasing sensitivity, specificity, and DOR of those mobile apps as participant age increased (Table 2).

For the eye chart selection, based on our literature review, Peek Acuity (Tumbling E chart) [33,34], Sightbook app (Snellen chart), and Eye Chart Pro app (Snellen chart) are the most commonly used methods for testing VA. Notably, the apps with a fast-testing algorithm completed VA tests with greater reliability and precision. Examiners should select age-appropriate standardized charts, randomize letters or optotypes, vary screen illumination, adjust the size of letters or optotypes, and store or transmit the VA data collected. Compared with smartphones, tablets have larger screens with higher resolutions able to display an assortment of letters and optotype (test symbol) charts at both high and low contrasts. However, we found that VA testing via smartphones have better performance than those via iPads. Nik Azis et al [21] used iPads to test VA of children aged 5 to 6 years with a 3-meter distance using the Lea symbols chart. The other 5 studies using smartphones [13,17,19,20,22] tested the participants with 2-meter distance using the Tumbling E chart. It is uncertain if the differences in test performance between apps for iPad and apps for smartphones are caused by the test distance. Therefore, using different VA charts with different testing distances may influence the performance of iPad-based apps. Further studies are needed to identify the cause of variable VA testing performance.

Early identification and management of children with VI is important [35] as nearly 19 million children in the world live with this condition. The World Health Organization suggests that refractive errors are one of the most common causes of these impairments, especially for children in low-income countries [4]. Mobile apps that provide VA tests can address this issue and make VA testing easier. In our 6 reviewed meta-analyses, 5 concentrated on children’s vision screening [17,19-22]. Among those studies, 4 [17,19,20,22] tested VA using Android smartphones with the Peek Acuity app (Tumbling E chart). We found a good correlation between VA via Peek Acuity and clinical standard examination. However, our analysis results showed lower specificity for children aged 3 to 5 years than the other 2 age groups. Previous literature has reported that the test of resolution acuity (eg, Tumbling E) may overestimate VA and thus be less sensitive for ocular diseases than tests of recognition acuity (eg, Lea, HOTV, Snellen) [26]. The American Association for Pediatric Ophthalmology and Strabismus Vision Screening app (ie, Lea symbols chart) was evaluated by Nik Azis et al [21] via an iPad mini in Malaysia among children aged 5 to 6 years.

Accurate tests of VA can be performed by nonprofessional examiners using a mobile VA app. Professional training in vision screening may be one of the factors affecting the accuracy and reliability of VA testing. However, our meta-analysis reveals that the nonprofessional examiner group had higher accuracy (AUC 0.93) than the professional examiner group (AUC 0.87), especially for children. Given children’s limited psychological and cognitive aptitude, parents or school teachers may better understand their children’s responses, behavior, and mood than eye care professionals. Thus, parents or school teachers can potentially be eye screeners to test children’s VA via mobile apps. For older adults, VA may be tested at patients’ homes by an eye care worker with basic training or a field worker without formal training. For example, in Bastawrous et al [13], Peek Acuity was used at patients’ homes by a community health care worker and achieved 84.6% sensitivity and 97.7% specificity in detecting eyes with severe VI.

### Limitations and Future Research

We recognize that this study has several limitations that provide opportunities for future research. First, the findings of this study should be interpreted with caution considering only a small number of studies were included in the meta-analysis. To increase the generalizability of our findings, more studies with longer follow-up periods are needed. Second, among the selected meta-analyses, there were no studies involving participants aged 22 to 54 years. Only one study involved participants aged 55 years and older [13], limiting the generalizability of the findings about that age group. Third, only one meta-analysis focused on VA tests using an iPad [21], which may not be representative. Thus, in subgroup analysis, we could not plot the summaries of sensitivity and specificity in Figure 5 and SROC curves in Figure 3. Fourth, some selected studies did not apply any strict standardized VA examination process and conditions. For instance, the brightness of a mobile device’s screen could not be adjusted to be precisely the same as the light-box chart [9]. Some studies evaluated the sensitivity and specificity of VA of left and right eyes separately. For example, Nik Azis et al [21] demonstrated that right vision screening had a higher sensitivity and specificity than left vision screening.

Future research should explore how to determine abnormal changes in eye anatomy and functions. Multifunctional vision evaluation apps that integrate digital eye image recognition techniques may make smartphones and tablets more attractive for ophthalmic assessment. The modern artificial intelligence and mHealth techniques have great potential in improving timely VI detection and treatment.

### Conclusions

The findings of this study suggest that mobile VA test apps can play an important role in identifying VI by professional examiners as well as nonprofessionals, who can perform self-testing at a time and place convenient to them. Public awareness of the safety and benefits of VA tests should be promoted, and further research with a larger sample and longer follow-up are needed to evaluate the potential role of a mobile phone VA test app.

## Appendix

Multimedia Appendix 1 Database search strategies.

Multimedia Appendix 2 Main study characteristics and findings from 8 studies that examined visual acuity by iPad apps.

Multimedia Appendix 3 Main study characteristics and findings from 8 studies that examined visual acuity by smartphone apps.

Multimedia Appendix 4 Multivariate meta-regression (sensitivity) for mobile device–based app in evaluating the visual acuity.

Multimedia Appendix 5 Multivariate meta-regression (specificity) for mobile device–based app in evaluating the visual acuity.

## Abbreviations

AUC: area under the curve
DOR: diagnostic odds ratio
ETDRS: Early Treatment Diabetic Retinopathy Study
logMAR: Logarithm of the Minimum Angle of Resolution
LR+: likelihood ratio positive
LR–: likelihood ratio negative
mHealth: mobile health
QUADAS-2: Quality Assessment of Diagnostic Accuracy Studies–2
SROC: summary receiver operating characteristic
VA: visual acuity
VI: vision impairment
