# Supplementary material for: Use of Mobile Apps for Visual Acuity Assessment: Systematic Review and Meta-analysis
Source: JMIR Mhealth Uhealth. 2022 Feb 14;10(2):e26275. doi: 10.2196/26275 (PMC8887635; doi:10.2196/26275)
Supplement: Multimedia Appendix 1 [file mhealth_v10i2e26275_app1.docx]

Multimedia Appendix 1, Table S1. Database search strategies:

| Database | Search strategy |
| --- | --- |
| PubMed | ("smartphone"[MeSH Terms] OR "smartphone"[All Fields] OR "smartphones"[All Fields] OR "smartphone's"[All Fields] OR "iphone"[All Fields] OR "iphones"[All Fields] OR "phone's"[All Fields] OR "phones"[All Fields] OR "telephone"[MeSH Terms] OR "telephone"[All Fields] OR "phone"[All Fields] OR "mobile"[All Fields] OR "mobiles"[All Fields] OR "mobile devices"[All Fields] OR "application"[All Fields] OR "applications"[All Fields]) AND ("Snellen chart"[All Fields] OR "Snellen's chart"[All Fields] OR "E chart"[All Fields] OR "visual acuity"[MeSH Terms] OR ("visual"[All Fields] AND "acuity"[All Fields]) OR "visual acuity"[All Fields]) AND ("test"[All Fields] OR "screen"[All Fields] OR "screening"[All Fields]) |
| Embase | ('smartphone' OR 'iphone' OR 'ipad' OR 'phone' OR 'mobile' OR 'mobile devices' OR 'app' OR 'application') AND ('snellen chart' OR 'e chart' OR 'visual acuity testing' OR 'va testing' OR 'eye screening') |
| Cochrane Library | ('smartphone' OR 'iphone' OR 'ipad' OR 'phone' OR ‘phone’/exp OR 'mobile' OR 'mobile devices' OR 'app' OR 'application') AND ('snellen chart' OR 'e chart' OR 'visual acuity' OR 'VA') AND (‘test’ OR ‘screen’ OR ‘screening’) |
| Google Scholar | ('smartphone' OR 'iphone' OR 'ipad' OR 'phone' OR ‘phone’/exp OR ‘telephone’ OR ‘telephone’/exp OR 'mobile' OR 'mobile devices' OR 'app' OR 'application') AND ('snellen chart' OR 'e chart' OR 'visual acuity' OR 'VA') AND (‘test’ OR ‘screen’ OR ‘screening’) |
