# Supplementary material for: Use of Mobile Apps for Visual Acuity Assessment: Systematic Review and Meta-analysis
Source: JMIR Mhealth Uhealth. 2022 Feb 14;10(2):e26275. doi: 10.2196/26275 (PMC8887635; doi:10.2196/26275)
Supplement: Multimedia Appendix 2 [file mhealth_v10i2e26275_app2.docx]

Multimedia Appendix 2, Table S1. Main study characteristics and findings from 8 studies that examined visual acuity by iPad apps

| Source | Study Design | Age, year | Sample size (P/E) | iPad type | App name | App description | TD | Main results |
| --- | --- | --- | --- | --- | --- | --- | --- | --- |
| Zhang et al., 2013[8], China | Observational study | 47 | 120/240 | iPad 2 | Eye Chart Pro app | Tumbling E chart | 2.5m | iPad is reliable when the Snellen VA better than 0.1. |
| Black et al., 2013[1], New Zealand | Observational study | 28 | 85 | iPad 1 | Bailey-Lovie chart | ETDRS chart | 6m | The iPad did not differ from the gold standard ETDRS chart (p>0.05) |
| Phung et al., 2016[5], USA | Observational study | 65.8 | 126/148 | iPad 1 | SightBook | Snellen chart | 14 inches | The discrepancy between SightBook mobile app and the clinic charts acuities is large |
| O’Neill, and McAndrew, 2016[4], Australia | Prospective quantitative comparative study | ≥18 | 60/120 | iPad2/iPhone 4 | Eye Chart Pro | Snellen chart | 2.5m  /1.2m | High level of general agreement between testing modality (ICC  0.917 with a 95% confidence interval of 0.887–0.940) |
| Rhiu et al., 2016[6], Korea | Observational study | 30 | 43/86 | iPad 3 | Mirroring chart | Snellen/Tumbling E/Landolt C/Numbers | 4m | The logMAR VA showed no significant difference between the ETDRS chart and the iPad Snellen chart (p = 0.66) and iPad Arabic figure chart (p = 0.29). |
| Kergoat et al., 2017, [3], Canada | Observational study | ≥65 | 150 | iPad | N/A | Letters/Numbers/Tumbling E | 3 m / 40 cm | 14.7% did not respond to any optotype, while 85.3% responded to letters, 84.0% to numbers, and 66.0% to tumbling E’s |
| Fernández et al., 2019[2], Spain | Observational study | 59.7 ± 8.5, | 59 | iPad | defocus curve app | ETDRS chart | 2 m | A total of 45.8% of eyes showed no differences between both tests and the difference was less than one line of VA in 96.6% of the eyes |
| Rodríguez-Vallejo Met et al., 2016[7], Spain | Observational study | 36 ± 11 | 45 | iPad | FSVA | HOTV | 3m | Forty-five healthy subjects with monocular corrected visual acuities better than 0.2 logMAR participated in the agreement study. |

P/E=participant/eye, TD=test distance, m=meter.

**References:**

1 Black JM, Jacobs RJ, Phillips G, Chen L, Tan E, Tran A, Thompson B (2013) An assessment of the iPad as a testing platform for distance visual acuity in adults. Bmj Open 3: e2730 Doi 10.1136/bmjopen-2013-002730

2 Fernández J, Rodríguez-Vallejo M, Tauste A, Albarrán C, Basterra I, Piñero D (2019) Fast Measure of Visual Acuity and Contrast Sensitivity Defocus Curves with an iPad Application. The Open Ophthalmology Journal 13: 15-22 Doi 10.2174/1874364101913010015

3 Kergoat H, Law C, Chriqui E, Leclerc B, Kergoat M (2017) Tool for Screening Visual Acuity in Older Individuals With Dementia. American Journal of Alzheimer's Disease & Other Dementiasr 32: 96-100 Doi 10.1177/1533317517689877

4 O'Neill S, McAndrew DJ (2016) The validity of visual acuity assessment using mobile technology devices in the primary care setting. Aust Fam Physician 45: 212-215

5 Phung L, Gregori NZ, Ortiz A, Shi W, Schiffman JC (2016) REPRODUCIBILITY AND COMPARISON OF VISUAL ACUITY OBTAINED WITH SIGHTBOOK MOBILE APPLICATION TO NEAR CARD AND SNELLEN CHART. Retina 36: 1009-1020 Doi 10.1097/IAE.0000000000000818

6 Rhiu S, Lee HJ, Goo YS, Cho K, Kim J (2016) Visual Acuity Testing Using a Random Method Visual Acuity Application. Telemed E-Health 22: 232-237 Doi 10.1089/tmj.2015.0073

7 Rodríguez-Vallejo M, Llorens-Quintana C, Furlan WD, Monsoriu JA (2016) Visual acuity and contrast sensitivity screening with a new iPad application. Displays 44: 15-20 Doi 10.1016/j.displa.2016.06.001

8 Zhang Z, Zhang S, Huang X, Liang L (2013) A Pilot Trial of the Ipad Tablet Computer as a Portable Device for Visual Acuity Testing. J Telemed Telecare 19: 55-59 Doi 10.1177/1357633X12474964
