# Supplementary material for: Use of Mobile Apps for Visual Acuity Assessment: Systematic Review and Meta-analysis
Source: JMIR Mhealth Uhealth. 2022 Feb 14;10(2):e26275. doi: 10.2196/26275 (PMC8887635; doi:10.2196/26275)
Supplement: Multimedia Appendix 3 [file mhealth_v10i2e26275_app3.docx]

Multimedia Appendix 3, Table S1. Main study characteristics and findings from 8 studies that examined visual acuity by smartphone apps

| Source | Study Design | Age, year | Sample size (S/E) | Smartphone type | App name | App description | TD | Main results |
| --- | --- | --- | --- | --- | --- | --- | --- | --- |
| Jan-Bond et al., 2015[2], Malaysia | Cross-sectional study | 37.0 ±15.9 | 101/202 | iPhone 4 | REST app | Tumbling E chart | 1 m/ 3m | Significant and strong direct correlation between VA using ETDRS and REST in both eyes (RE: r=0.829; p< 0.001, LE: r=0.871; p<0.001) |
| Lodha et al., 2016[4], India | Observational study | 46 | 92/46 | Android operating system based Smartphone | Interactive Visual Acuity Chart | 7 lines of English  alphabets of decreasing size | 2 m | 92.35% results matching the results obtained with standard Snellen chart at 6 meters distance |
| Han et al., 2019[3] China/Australia | Population-based study | 50–79 | 100/200 | iPhone 7 plus  (iOS11) | V@home | Standard ETDRS | 2m | High agreement of V@home with  near ETDRS VA across all groups, with a mean difference of -0.092 to -0.042 logMAR and a TWK of 0.736 to 0.837. |
| Tofigh et al., 2015[7], USA | N/A | 18–89 | 100/200 | iPhone 5 | EyeHandBook | Snellen chart | 36 cm | With P-value of <0.0001, compared with the conventional near vision card by an average of 0.11 LogMAR unless the measurement done by the near vision card was 20/20. |
| [6], Australia | Prospective comparative study | ≥16 | 88/88 | iPhone 4 | 11 apps (e.g., Eye Test, OptOK, etc.) | Snellen | 1.2m | Eleven applications were identified, with accuracy of optotype size ranging from 4.4–39.9%. |
| Toy et al., 2016[8], USA | Prospective study | 60.5 ± 10.6 | 50/100 | iPhone 5s | SightBook | Rosenbaum near chart | 14 inches | The correlation between clinical Snellen and smartphone visual acuity measurements is rho = 0.91). |
| Brady et al., 2015[1], Kenyan | Validation study | ≥55 | 300/600 | N/A | Peek Acuity | Tumbling-E chart | 4 m | The agreement of Peek Acuity and  the ETDRS chart was greater than the Snellen chart with the ETDRS chart (95%CI, 0.05-0.10; P = .08) |
| Pathipati et al., 2016[5]  , USA | Observational study | 48.5± 19.8 | 64/128 | iPhone | Paxos Checkup^TM^ | Sightbook | 20 feet | ED logMAR BCVA was 0.21 ± 0.35 (approximately 2 Snellen lines difference ± 3 Snellen lines) higher than that of ophthalmologists when ED staff used a Snellen chart (p = .0.00003). |

P/E=participant/eye, TD=test distance, m=meter.

**References:**

1 Brady CJ, Eghrari AO, Labrique AB (2015) Smartphone-Based Visual Acuity Measurement for Screening and Clinical Assessment. JAMA 314: 2682 Doi 10.1001/jama.2015.15855

2 Chan Jan-Bond TWNH (2015) REST AN INNOVATIVE RAPID EYE SCREENING TEST. Journal MTM 4:3:2025

3 Han X, Scheetz J, Keel S, Liao C, Liu C, Jiang Y, Müller A, Meng W, He M (2019) Development and Validation of a Smartphone-Based Visual Acuity Test (Vision at Home). Transl Vis Sci Techn 8: 27 Doi 10.1167/tvst.8.4.27

4 Lodha VS (2015) Comparison of visual acuity measurement by Smartphone based application vs. conventional Snellen visual acuity chart. International Journal of Enhanced Research in Medicines & Dental Care Vol. 2 Issue 6: 39-41

5 Pathipati AS, Wood EH, Lam CK, Sales CS, Moshfeghi DM (2016) Visual acuity measured with a smartphone app is more accurate than Snellen testing by emergency department providers. Graefes Arch Clin Exp Ophthalmol 254: 1175-1180 Doi 10.1007/s00417-016-3291-4

6 Perera C, Chakrabarti R, Islam FM, Crowston J (2015) The Eye Phone Study: reliability and accuracy of assessing Snellen visual acuity using smartphone technology. Eye (Lond) 29: 888-894 Doi 10.1038/eye.2015.60

7 S Tofigh ESAE (2015) Effectiveness of a smartphone application for testing near visual acuity. Eye

8 Toy BC, Myung DJ, He L, Pan CK, Chang RT, Polkinhorne A, Merrell D, Foster D, Blumenkranz MS (2016) SMARTPHONE-BASED DILATED FUNDUS PHOTOGRAPHY AND NEAR VISUAL ACUITY TESTING AS INEXPENSIVE SCREENING TOOLS TO DETECT REFERRAL WARRANTED DIABETIC EYE DISEASE. Retina 36: 1000-1008 Doi 10.1097/IAE.0000000000000955
