# Supplementary material for: Use of Mobile Apps for Visual Acuity Assessment: Systematic Review and Meta-analysis
Source: JMIR Mhealth Uhealth. 2022 Feb 14;10(2):e26275. doi: 10.2196/26275 (PMC8887635; doi:10.2196/26275)
Supplement: Multimedia Appendix 5 [file mhealth_v10i2e26275_app5.docx]

| Multimedia Appendix 5, Table S1. Multivariate meta-regression (specificity) for mobile device-based app in evaluating the visual acuity | | | | | | |
| --- | --- | --- | --- | --- | --- | --- |
|  |  |  |  |  |  |  |
| Variables | No. of study | Coefficient | Lower 95% CI | Upper 95% CI | *P* |  |
|  |  |  |  |  |  |  |
| Year of publication | 8 | 0.006 | -0.18 | 0.2 | 0.102 |  |
| Sample size | 8 | 8.60×10^6^ | -0.00002 | 0.00004 | 0.402 |  |
| Mobile device | 8 |  |  |  |  |  |
| smartphone | 6 | Reference | | | |  |
| iPad | 2 | -0.02 | -0.26 | 0.22 | 0.834 |  |
| Examine | 8 |  |  |  | 0.99 |  |
| Test by Professional examiner | 4 | Reference | | | |  |
| Test by non-Professional examiner | 4 | -0.001 | -0.25 | 0.25 | 0.808 |  |
| CI, confidence interval | | | | | | |
